# Oral Function, Frailty and Mortality in Older Adults: Evidence from the Chilean National Health Survey 2016–2017

**DOI:** 10.3390/ijerph23040538

**Published:** 2026-04-21

**Authors:** Gustavo Sáenz-Ravello, Mauricio Baeza, Laura Sáenz-Ravello, Carol Guarnizo-Herreño, Jorge Gamonal

**Affiliations:** 1 Center for Epidemiology and Surveillance of Oral Diseases (CESOD), Faculty of Dentistry, University of Chile, Santiago 7530263, Región Metropolitana, Chile; mbaeza.paredes@odontologia.uchile.cl (M.B.); jgamonal@odontologia.uchile.cl (J.G.); 2 Consultorio General Rural de Camiña, Servicio de Salud Tarapacá, Camiña 1150000, Región de Tarapacá, Chile; 3 School of Health and Wellbeing, Health Economics and Health Technology Assessment, College of Medical Veterinary and Life Sciences, University of Glasgow, Glasgow G12 8QQ, UK; 4 Departamento de Gestión Odontológica, Servicio de Salud Tarapacá, Ministerio de Salud, Anibal Pinto 815, Iquique 1100781, Región de Tarapacá, Chile; 5 School of Nursing, Facultad de Medicina, Pontificia Universidad Catolica de Chile, Santiago 7820436, Región Metropolitana, Chile; laura.saenz@estudiante.uc.cl; 6 Departamento de Salud Colectiva, Facultad de Odontología, Universidad Nacional de Colombia, Bogotá 111321, Colombia; ccguarnizoh@unal.edu.co; 7 Interuniversity Center for Healthy Aging RED21993, Santiago 7530263, Región Metropolitana, Chile

**Keywords:** oral health, frailty, mortality, aged

## Abstract

**Highlights:**

**Public health relevance—How does this work relate to a public health issue?**

- Population ageing in Chile is occurring alongside a high burden of frailty, multimorbidity, and functional decline, yet oral health remains underrecognized within healthy ageing and mortality research.
- Using nationally representative Chilean survey data linked to mortality records, this study examines whether oral functional status—captured through minimum dentition and denture use—is associated with all-cause mortality in older adults.

**Public health significance—Why is this work of significance to public health?**

- Older adults with more favorable oral functional-status categories showed lower all-cause mortality hazards than those with fewer than 10 teeth and no prostheses, even after adjustment for age, sex, education, area of residence, frailty, diabetes, and hypertension.
- These findings support the view that oral function may act as a marker of broader health vulnerability in later life and strengthen the rationale for integrating oral health into population-based ageing research in Latin America.

**Public health implications—What are the key implications or messages for practitioners, policy makers and/or researchers in public health?**

- Oral functional measures such as minimum dentition and prosthesis use may be useful additions to healthy ageing surveillance, risk stratification, and public health assessment of older populations.
- Because the analysis is exploratory and based on a modest analytic sample, larger longitudinal studies with repeated oral assessments and standardized outcome definitions are needed before drawing causal or policy-directive conclusions.

**Abstract:**

Background: Oral health is an often-overlooked component of healthy ageing, as a sign of cumulative functional decline. This study explored the association between oral functional status and all-cause mortality. Methods: This was a secondary analysis of the 2016–2017 Chilean National Health Survey linked to mortality records through December 31, 2022. In total, 223 participants aged ≥ 60 years were included (*N* = 1,016,557). Minimum dentition (MD) was defined as ≥10 teeth, prosthesis use was self-reported, and frailty was assessed using a modified Fried phenotype. Survey-weighted Cox models estimated associations with all-cause mortality, adjusting for age, sex, area, education, frailty, diabetes, and hypertension. Results: In the survey-weighted Cox model, each category representing MD and/or prosthesis use was associated with lower mortality hazards compared with the reference group (<10 teeth and no prostheses): prostheses only (HR 0.17, 95% CI 0.05–0.61), MD only (HR 0.16, 95% CI 0.04–0.74), and MD with prostheses (HR 0.08, 95% CI 0.01–0.46). Increasing age and rural residence were associated with higher mortality hazards, whereas estimates for sex, education, frailty, diabetes and hypertension were imprecise and generally compatible with no clear association. Conclusions: These findings are hypothesis-generating and support further evaluation of oral functional status as a marker of broader health vulnerability in aged Chileans.

## 1. Introduction

Population ageing is accelerating worldwide, particularly in middle-income countries, where gains in life expectancy increasingly coexist with a high burden of noncommunicable diseases [1], functional decline [2], and frailty [3]. In this context, mortality in older adults is shaped not only by the presence of specific diagnoses, but also by cumulative impairments in physiological reserve and functional capacity [4], which reflect long-term exposures to biological, behavioral, and social determinants of health [5,6].

Oral health is a highly relevant [7] yet often overlooked component of healthy ageing [8]. Tooth loss remains common among older adults and represents a cumulative marker of lifelong oral disease [7], access to care [9], and socioeconomic disadvantage [10,11]. Beyond its local consequences, tooth loss has been associated with adverse systemic outcomes, including malnutrition, systemic inflammation, cardiometabolic disease [12], and increased mortality [13,14]. In this sense, periodontitis, the primary cause of tooth loss in adults, may contribute to sustained low-grade systemic inflammation through recurrent bacteremia and elevated circulating inflammatory mediators, which in turn have been implicated in endothelial dysfunction, insulin resistance, and atherosclerotic processes [15]. Moreover, it has been suggested that tooth loss may serve as an early marker of frailty and general decline in older adults [16], as it reflects cumulative exposure to individual biology and lifestyle factors, living/working conditions, and societal policies that may impact trajectories of physiological reserve depletion and functional vulnerability. However, the clinical relevance of tooth loss may depend less on the absolute number of remaining teeth than on whether oral function is preserved.

The concept of minimum dentition or functional dentition has been proposed to capture this distinction, particularly defined as the presence of at least 10 natural teeth, sufficient to maintain basic masticatory function [17]. Loss of functional dentition has been linked to impaired dietary intake, reduced protein consumption, sarcopenia, and frailty pathways that plausibly connect oral health to survival in later life [18,19]. Dental prostheses or dentures may restore oral function in individuals with substantial tooth loss [20,21], yet evidence remains inconsistent as to whether prosthetic rehabilitation mitigates the excess mortality risk associated with tooth loss [22].

Importantly, oral health in older adults is embedded within a broader clinical context frequently characterized by multimorbidity and functional vulnerability [23]. Frailty, diabetes, and hypertension are highly prevalent in this population and may confound or modify associations between oral status and mortality. To our knowledge, few population-based studies have simultaneously examined functional dentition, prosthesis use, and mortality while accounting for these interrelated conditions [24,25], particularly in Latin American settings.

Using nationally representative data from the 2016–2017 Chilean National Health Survey (NHS) linked to official mortality records, this study explored the association between oral function and all-cause mortality among older adults, while accounting for sociodemographics, frailty and major cardiometabolic conditions.

## 2. Materials and Methods

This study was a secondary analysis of epidemiological data obtained from a NHS conducted in 2016–2017. This report follows the STROBE guidelines for cohort studies [26].

### 2.1. National Health Survey 2016–2017

**Figure A1:**
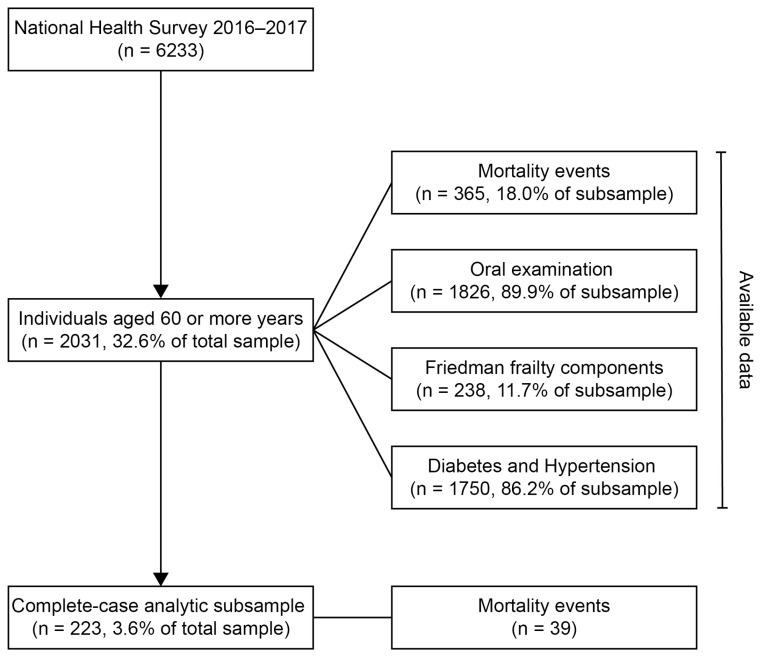
Derivation of the complete-case analytic sample and availability of key variables among eligible older adults (≥60 years) in the 2016–2017 Chilean National Health Survey. The left column shows the sequential derivation of the analytic sample; the right column shows the parallel availability of key variables among eligible older adults. The final analytic sample corresponds to the intersection of participants with complete data for all variables required in the primary analysis.

The NHS was a cross-sectional, probabilistic, population-based survey, stratified by conglomerate (multi-stage), whose target population was persons aged 15 years and older who usually reside in privately occupied housing, located in the urban and rural areas of 15 regions of Chile. Thirty strata were formed by crossing regions and locations, including municipalities with more than 30,000 inhabitants, to select census blocks (urban areas) or localities (rural areas), dwellings, and one individual at random per dwelling. A total sample of 6233 respondents aged 15 years and over was obtained, which, utilizing the survey weights, corresponded to an expanded sample of *N* = 14,518,969 individuals. The non-response rate of was 9.8%. Interviews were conducted at home, using a tablet, by an interviewer or nurse according to the type of form. The fieldwork was done between August 2016 and March 2017. In addition, a random subgroup of 5520 individuals underwent laboratory tests, with a sampling error of 2.6% nationwide due to the design effect of 1797 estimates with 95% confidence and a relative error of <30%. The broad age range of the NHS was a design feature of the original survey, established by the Ministry of Health, and was not under the control of the investigators of the present study. The analytic sample of this study was restricted to participants aged 60 years and older. To improve comparability across sequential model specifications, the model-sequence analysis was restricted to a fixed complete-case subsample with non-missing values for all covariates considered across candidate models (oral functional-status categories, sex, age, educational attainment, area of residence, frailty, diabetes, and hypertension). Among 2031 individuals aged 60 or more, a total of 223 participants aged 60 or more years were included (Figure A1).

The project involved in the NHS execution was approved by the Scientific Ethical Committee of the Faculty of Medicine of the Pontificia Universidad Católica de Chile (project no. 16-019). Informed consent was obtained from the population aged ≥ 18 years. Assent and informed consent from a representative were required for those aged under 18. For detailed information on the evaluation methods, please visit the following webpage http://epi.minsal.cl/encuesta-ens-descargable/ (accessed on 17 January 2026).

### 2.2. Study Measures

#### 2.2.1. Sociodemographic Data

Age (continuous), sex (male/female), place of residence (urban/rural), and educational attainment (years of schooling approved and categorized as <8, 8–12, and ≥13 years) were recorded.

#### 2.2.2. Oral Health

Nurses received standardized training delivered by nine dentists, which included a demonstration session, supervised practice of dental examinations, and a final assessment consisting of 20 clinical cases. The mean test score for nurse’s assessment of oral health was 50.0 (SD 2.7), and inter-rater reliability was substantial (Cohen’s kappa = 0.85). The oral health assessment collected information on prostheses use and total number of remaining teeth (range: 1–16 per arch). Presence of missing anterior teeth, and cavitated carious lesions in both the maxilla and mandible were also recorded; however, these variables were not included in the analytic models of the present study. Additionally, the questionnaire recorded whether anterior tooth loss was functionally compensated by prostheses use. Detailed information regarding the oral health evaluation procedures is available at the Chilean NHS website (http://epi.minsal.cl/encuesta-ens-descargable/, accessed on 17 January 2026). Minimum dentition was defined as the presence of at least 10 natural teeth in total (minimum of five teeth per arch, without considering their location or whether they constitute occlusal pairs) and coded as a binary variable, according to previous studies [13,27]. This operationalization was constrained by the survey data, which recorded the total number of teeth per arch but not individual tooth positions or occlusal contacts. Prosthesis use was defined as a binary variable indicating self-reported use of a dental prosthesis (maxillary, mandibular or both).

#### 2.2.3. Frailty

Frailty was assessed using a modified version of the Fried frailty phenotype, incorporating five domains: muscle strength, physical activity, gait speed, nutritional status (body mass index, BMI), and functional capacity [28]. Low muscle strength was defined as handgrip strength below the 20th percentile of the population distribution. Handgrip strength was measured bilaterally using a standardized dynamometry protocol, and the maximum value obtained was used for analysis (kg). Physical inactivity was defined as engaging in less than 150 min per week of physical activity and was further operationalized using the Global Physical Activity Questionnaire (GPAQ), with inactivity defined as a total energy expenditure below 600 MET-minutes per week [29]. Slow walking speed and difficulty performing activities of daily living were assessed by self-report and categorized as “I have no problems; I have some problems performing and I am unable to perform my usual activities”. Body weight and height were measured using standardized procedures and used to calculate the BMI, with low BMI defined as <23.0 kg/m^2^ according to age-specific cut-offs [30]. Following a classical interpretation of the frailty phenotype, participants presenting three or more of these five criteria were classified as frail, those presenting one or two criteria as pre-frail, and those with none of the criteria as robust [31]. For the regression analyses conducted in this study, frailty status was additionally dichotomized as frailty (pre-frail and frail) versus non-frail (robust).

#### 2.2.4. Type 2 Diabetes Mellitus

Diabetes status was defined using a combined algorithm based on self-reported diagnosis and fasting blood glucose levels. Self-reported diabetes was identified through a positive response to a survey question, indicating a previous diagnosis of diabetes by a health professional. Biochemical diabetes was defined as a fasting plasma glucose level ≥ 126 mg/dL, measured after a fasting period of at least 8 h. Individuals who reported a history of gestational diabetes were excluded from the diabetes classification algorithm. Participants who reported that they did not remember or were unsure whether a health professional had ever diagnosed them with diabetes were treated as missing for this variable. This combined approach was used to capture both diagnosed and previously undiagnosed diabetes cases in the study population.

#### 2.2.5. Hypertension

Hypertension status was defined using a combined algorithm based on self-reported treatment and measured blood pressure values. Self-reported hypertension was identified through affirmative responses to questions about current treatment, defined as the use of antihypertensive medications or combined treatment. In addition, measured hypertension was defined as a mean systolic blood pressure ≥ 140 mmHg and/or a mean diastolic blood pressure ≥ 90 mmHg, calculated from the average of three standardized blood pressure measurements. Participants meeting either the self-reported treatment criteria or the measured blood pressure thresholds were classified as having hypertension.

#### 2.2.6. Mortality

Mortality data were obtained from the official national mortality registry managed by the Chilean Ministry of Health, which compiles death records from the Civil Registry. NHS records were deterministically linked to mortality data using a unique anonymized individual identifier derived from the national identification number (RUT), allowing accurate ascertainment of vital status and cause of death while preserving participant confidentiality. No personally identifiable information was accessible to the investigators. Date of events (follow-up) were recorded until 31 December 2022. Cause-of-death information (ICD-10 underlying cause) was summarized descriptively among deaths occurring in the analytic sample.

### 2.3. Statistical Analysis

All analyses accounted for the complex survey design of the NHS, including sampling weights, stratification, and clustering, using the survey procedures in Stata 19.5 SE (StataCorp. 2025. StataCorp LLC., College Station, TX, USA). Descriptive statistics were calculated using survey-weighted estimates and are presented as means (or medians) or proportions with confidence intervals at 95%, as appropriate for the data distribution. Group comparisons for categorical variables were performed using the survey-adjusted chi-square or Fisher’s exact test, and ANOVA or Kruskal–Wallis tests for continuous variables. Survival time was defined as the interval between the survey beginning date and the date of death or censoring, expressed in years. The primary outcome was all-cause mortality.

**Figure 1:**
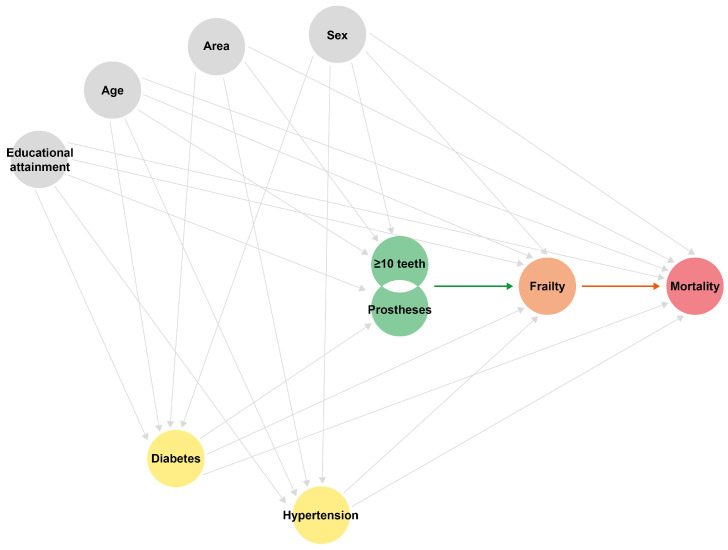
Directed acyclic graph (DAG) illustrating the hypothesized relationships between oral functionality, frailty, noncommunicable diseases, and mortality.

**Figure A2:**
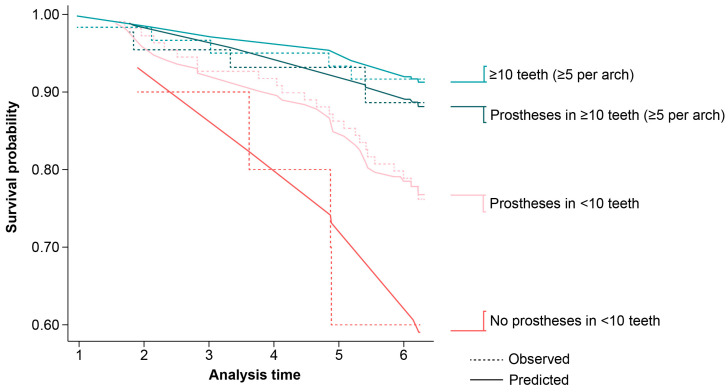
Predicted and observed survival probabilities according to minimum dentition and prostheses.

Associations between oral functional status and all-cause mortality were estimated in an exploratory framework using survey-weighted Cox proportional hazards regression models. Minimum dentition and prosthesis use were modelled as the main exposures, namely, oral function. Sequential models were used to examine directional consistency and estimate stability across prespecified adjustment sets, rather than to make formal model-selection claims. Models were sequentially adjusted for social determinants of health namely age (continuous), sex, area of residence (urban/rural) [32], educational attainment; frailty status, as it captures cumulative physiological decline and functional vulnerability that may confound the association between oral status and mortality [33,34]. Frailty was included as a covariate under a conservative analytic approach. However, given evidence that poor oral health may itself contribute to frailty development [35,36], frailty could also operate as an intermediate factor on the causal pathway (results are presented sequentially with and without frailty adjustment, to assess the potential impact of this dual role). Type 2 diabetes and hypertension were included as prevalent cardiometabolic conditions independently associated with both poor oral health and increased mortality risk [37,38]. Covariates were selected a priori based on clinical relevance and biological plausibility to construct a directed acyclic graph (DAG) (Figure 1). No data-driven variable selection or screening based on univariable statistical significance was performed. Proportional hazards assumptions were assessed graphically and found to be likely acceptable (Figure A2).

As an exploratory sensitivity analysis, we fitted a generalized structural equation model aligned with the prespecified DAG to assess whether the direction of associations was qualitatively consistent across jointly modelled pathways. The model jointly specified equations for oral function (binary outcomes, logit link), frailty status (binary outcome, logit link), cardiometabolic conditions (diabetes and hypertension, binary outcomes, logit link), and time to death (parametric survival model with a Weibull distribution). The integration method for the random-effects model was Laplacian approximation with 7 squaring points. Coefficients were reported with a 95% CI based on a linearized error estimate for survey data.

Unadjusted survival curves were estimated using Kaplan–Meier methods for descriptive purposes. Adjusted survival curves were generated from the Cox regression models to illustrate predicted survival according to combinations of minimum dentition and prosthesis use, while holding covariates constant. Hazard ratios (HRs) with 95% confidence intervals (CIs) are reported. Two-sided *p*-values are reported for descriptive inference. Interpretation focused primarily on effect estimates, confidence intervals, and directional consistency across model specifications. *p* < 0.05 was used as a conventional reference threshold.

## 3. Results

A total of 223 participants were included, representing a weighted population of 1,016,557 individuals (Table 1). The mean age of the study population was 69.63 years (95% CI: 68.02–71.23). Participants with <10 teeth tended to be older than those with ≥10 teeth (≥5 per arch), regardless of prosthesis status (mean age: 74.46 years [95% CI: 68.74–76.18] for <10 teeth without prostheses, 72.93 [70.41–75.46] for <10 teeth with prostheses, 66.37 [64.12–69.21] for ≥10 teeth without prostheses, and 67.44 [64.82–70.07] for ≥10 teeth with prostheses; *p* < 0.05). Sex distribution was balanced across groups, with no statistically significant differences (*p* > 0.1).

Educational attainment differed significantly across dental status groups (*p* < 0.01). Individuals with <10 teeth were more frequently represented in the lowest educational category (<8 years: 47.59% and 60.32% in those without and with prostheses, respectively), whereas participants with ≥10 teeth without prostheses showed the highest proportion with ≥13 years of education (44.60%). Most participants resided in urban areas (99.36%, 95% CI: 97.95–99.81), with no statistically significant differences by dental status (*p* > 0.1).

Frailty status did not differ significantly across groups (*p* > 0.1), although a descriptive pattern was observed. The proportion classified as frail was highest among participants with <10 teeth without prostheses (27.11%, 95% CI: 6.16–67.83) and lower among those with ≥10 teeth (7.88% without prostheses and 4.26% with prostheses), while the robust category was most frequent in the ≥10 teeth without prostheses group (41.82%, 95% CI: 27.92–57.15). The prevalence of diabetes did not differ significantly across dental categories (*p* > 0.1), although a descriptive trend was also observed. Diabetes prevalence was lower among individuals with ≥10 teeth without prostheses (17.61%) and higher in those with <10 teeth without prostheses (43.60%) and ≥10 teeth with prostheses (37.20%). Likewise, hypertension prevalence was high across all groups and was not statistically different (*p* > 0.05), although it was numerically higher among participants with <10 teeth (86.41% without prostheses; 77.52% with prostheses) than among those with ≥10 teeth (56.35% without prostheses; 55.52% with prostheses).

Among the 39 deaths with available cause-of-death classification, neoplasms were the leading cause of mortality, accounting for 38.5% of all deaths (Table 2). The most frequent cancer-related causes included malignant neoplasms with digestive and respiratory malignancies accounting for several of the recorded neoplasm deaths. Respiratory diseases represented the second most common category (25.6%), driven primarily by COVID-19 (15.4% of all deaths), followed by interstitial pulmonary diseases with fibrosis and pneumonia-related conditions. Cardiovascular diseases accounted for 17.9% of deaths and included ischemic heart disease, heart failure, hypertensive heart disease, and cerebrovascular events (including intracerebral hemorrhage and sequelae of stroke). Neurological disorders, including dementia and Alzheimer disease, comprised 5.1% of deaths. Digestive diseases and symptoms/ill-defined conditions were infrequent, each contributing 2.6% of total mortality. A limited number of injury-related causes were observed (7.7%), including traumatic vascular injury, femoral fracture, and toxic exposure to gases/fumes.

### 3.1. Kaplan–Meier Survival Analysis

**Figure 2:**
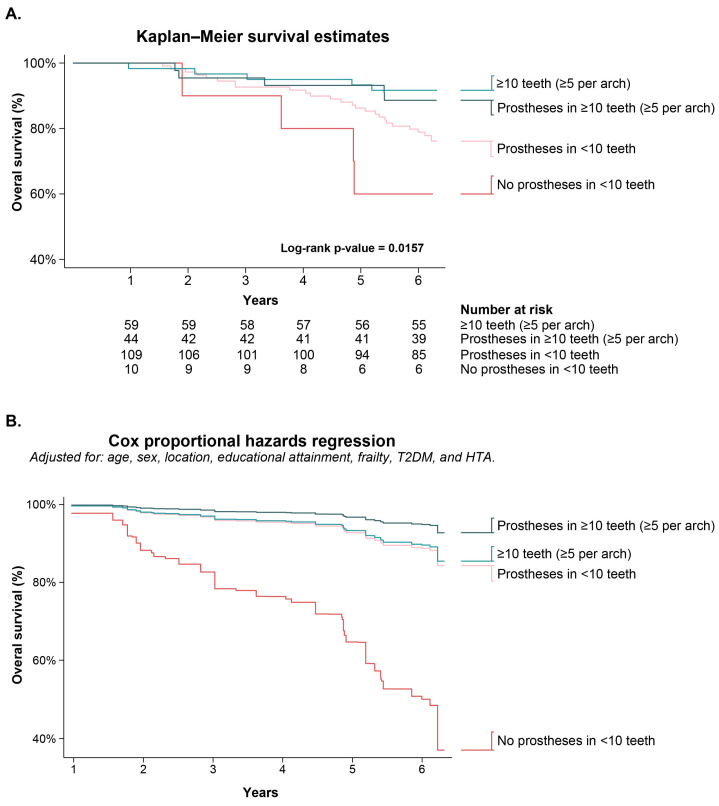
Survival analysis. (**A**). Kaplan–Meier curves depicting unadjusted overall survival probability according to minimum dentition and denture use. Differences between groups were assessed using the log-rank test. (**B**). Adjusted overall survival probabilities according to minimum dentition and denture use, derived from Cox proportional hazards regression models. Curves were adjusted for age, sex, place of residence (urban/rural), frailty status, type 2 diabetes mellitus (T2DM), and hypertension (HTA). Tick marks indicate censored observations.

Kaplan–Meier survival curves (Figure 2A) showed evidence of differences in overall survival according to minimum dentition and prosthesis use (log-rank *p* = 0.0157). Participants with ≥10 teeth tended to show higher survival probabilities over follow-up, whereas those with <10 teeth tended to show lower survival probabilities, particularly in the absence of prostheses.

### 3.2. Adjusted Cox Proportional Hazards Regression

**Figure 3:**
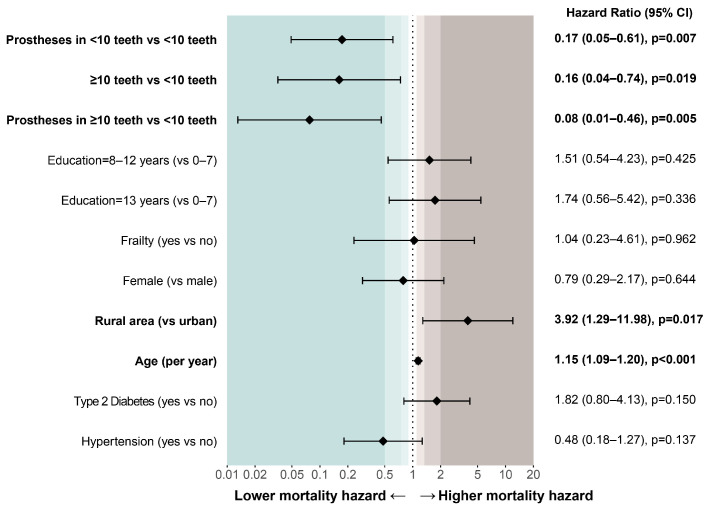
Forest plot showing log-scaled hazard ratios (HRs) and 95% confidence intervals (CIs) from the fully adjusted Cox proportional hazards regression model. The vertical dotted line denotes no association (HR = 1). HRs, 95% CIs, and *p*-values are shown on the right.

In sequential survey-weighted Cox models, the combined dentition/prosthesis categories showed directionally consistent inverse associations with all-cause mortality across prespecified model specifications (Table 3). Using participants with <10 teeth and no prostheses as the reference group, all other oral-status categories had hazard ratio estimates below 1.0 across models. In Model 3 (fully adjusted model), prosthesis use with <10 teeth was associated with lower all-cause mortality hazards (HR = 0.17, 95% CI: 0.05–0.61; *p* = 0.007), as was ≥10 teeth without prostheses (HR = 0.16, 95% CI: 0.04–0.74; *p* = 0.019) and ≥10 teeth with prostheses (HR = 0.08, 95% CI: 0.01–0.46; *p* = 0.005) (Figure 2B). Increasing age and rural residence were associated with higher mortality hazards. For sex, education, frailty, type 2 diabetes, and hypertension, estimates were generally imprecise and compatible with no clear association in this sample (Figure 3).

As an exploratory sensitivity analysis, a multivariable joint parametric survival model (generalized structural equation modelling with a Weibull time-to-event equation) yielded qualitatively similar directional patterns, with age showing the most precisely estimated positive association with all-cause mortality (Table A1). In the time-to-event equation, age was positively associated with all-cause mortality risk (β = 0.123, 95% CI: 0.074 to 0.172; *p* < 0.001). The coefficient for minimum dentition/prosthesis status was negative (β = −0.646, 95% CI: −1.350 to 0.057; *p* = 0.071), compatible with an inverse association but imprecise. Geographic area also showed an imprecise positive estimate (β = 1.385, 95% CI: −0.043 to 2.812; *p* = 0.057). Estimates for frailty status, sex, diabetes, hypertension, and educational attainment in the mortality equation were imprecise and included the null. In auxiliary equations, older age was associated with lower minimum dentition/prosthesis values and with hypertension, whereas educational attainment was positively associated with minimum dentition/prosthesis values and inversely associated with diabetes and frailty status.

## 4. Discussion

In this exploratory analysis of a nationally representative cohort of older adults in Chile, oral functional-status categories (based on dentition and prostheses use) were associated with subsequent all-cause mortality, with directionally consistent hazard ratio estimates across sequential survey-weighted Cox models. Participants with more favorable oral functional status categories tended to show lower mortality hazards than those with <10 teeth and no prostheses, even after adjustment for age, sex, area of residence, educational attainment, frailty, diabetes, and hypertension at baseline. However, given the modest analytic sample size and limited number of deaths, these findings should be interpreted as hypothesis-generating, and effect magnitudes should be viewed with caution. In particular, the reference group (<10 teeth without prostheses) comprised only 10 participants, rendering unstable point estimates of the hazard ratios (0.08–0.17). Accordingly, interpretation should focus on the consistent direction of the as-sociations across exposure categories and model specifications rather than on the magnitude of individual estimates.

The findings of this study are in line with evidence from large population-based cohorts showing that reduced oral function is associated with higher mortality. Analyses of NHANES data using clinically assessed number of teeth have reported increased all-cause mortality among individuals with fewer than 10 teeth, edentulism, or lack of functional dentition (HRs 1.3–1.5), even after extensive adjustment for sociodemographic and cardiometabolic factors [39]. Similarly, a propensity score–weighted survival analysis of partially edentulous adults demonstrated that removable partial prostheses use was associated with longer survival (event time ratio 1.26) and lower mortality rates compared with non-users [40]. In contrast, a smaller longitudinal study of community-dwelling older adults in Southern Brazil, defining functional dentition as ≥20 teeth and using Poisson models, reported no independent association with mortality after adjustment, underscoring the influence of exposure definitions, sample size, and analytic approach on observed associations [41]. While the limited number of events precludes cause-specific survival analyses, plausible mechanisms may link oral functional impairment to these outcomes [42]. Chronic periodontal disease and tooth loss have been associated with systemic inflammation and immune dysregulation, which may contribute to cancer susceptibility and progression [43]. Impaired masticatory function and dysphagia associated with tooth loss may increase the risk of aspiration pneumonia and respiratory complications [44,45], particularly among frail older adults. Additionally, the well-documented associations between periodontal disease, tooth loss, and atherosclerotic cardiovascular disease may partly explain the cardiovascular mortality observed [46]. It should be noted that the present analysis used standard Cox regression for all-cause mortality and did not employ a competing risks framework. Cause-specific approach could provide additional insight into whether oral functional status is differentially associated with specific causes of death [13,14].

An important conceptual question concerns whether frailty operates as a confounder, a mediator, or a marker of shared vulnerability within the oral health–mortality pathway. Recent meta-analytic evidence supports a predominantly intermediary role. Poor oral health, particularly having fewer than 20 teeth, has been consistently associated with increased risk of physical frailty [35,36], mediated through impaired mastication, malnutrition, and systemic inflammation, where prosthodontic rehabilitation alone appears insufficient to reverse nutritional decline without concurrent dietary counseling [47]. Concurrently, frailty and prefrailty have been robustly linked to elevated all-cause mortality [37,38,48]. These findings collectively support a sequential pathway in which tooth loss accelerates frailty, which in turn amplifies mortality risk. However, bidirectional mechanisms cannot be excluded, as sarcopenia and dysphagia may themselves worsen oral health [36], implying that frailty also partially acts as a confounder. In the present analysis, including frailty as a covariate in the fully adjusted model may have partially attenuated the oral health–mortality association if frailty lies on the causal pathway.

In addition, tooth loss has been proposed as a marker of accelerated aging trajectories, encompassing not only physical but also cognitive and functional decline. Prospective cohort studies have reported associations between severe tooth loss or edentulism and increased risk of sarcopenia, cognitive impairment, and all-cause mortality, particularly in middle-aged and older adults, suggesting that cognitive decline may represent an intermediate pathway linking compromised oral health to adverse survival outcomes [49,50]. However, evidence regarding cognition as an independent mediator of mortality remains mixed. While tooth loss has been associated with cognitive decline in general populations [49], studies among individuals with established dementia have not consistently demonstrated an independent association between tooth loss and subsequent mortality after robust covariate adjustment [51]. Taken together, these findings suggest that cognitive impairment may reflect a shared vulnerability or accelerated aging phenotype associated with tooth loss [52], rather than a direct causal pathway to mortality. On the other hand, these findings suggest that prosthesis use was associated with lower mortality hazards across oral-status categories, although estimates were imprecise and should be interpreted cautiously. They also remain compatible with the possibility that prostheses do not fully compensate for the loss of natural teeth. This pattern is consistent with prior literature suggesting that natural dentition provides biomechanical, sensory, and nutritional advantages that may be only partially restored by prosthetic devices [53,54].

### Strengths and Limitations

The use of nationally representative survey data linked to complete mortality records enhances the external validity of this study within the Chilean older adult population. However, caution is warranted when extrapolating these results to settings with different oral healthcare systems, higher baseline prosthetic coverage, or markedly different socioeconomic gradients. In addition, the findings may not apply to institutionalized older adults, who were not included in the survey sampling frame and may exhibit different patterns of oral health, frailty, and mortality risk [55].

The principal methodological limitation of this study is the substantial reduction from 2031 eligible older adults to 223 complete cases (approximately 11%), which constrained statistical power, particularly for detecting interaction effects and modest associations, and is reflected in the width of several confidence intervals. This reduction was driven primarily by the original survey’s random subsampling for laboratory and clinical assessments, a design feature that approximates a missing-completely-at-random mechanism, rather than by differential non-response [56]. In addition, the complete-case approach may have introduced selection bias, as participants with complete data may represent a healthier subset of the eligible population, potentially limiting the generalizability of the findings. Nevertheless, mortality ascertainment was complete through deterministic linkage to national death records, meaning that the outcome did not influence the probability of being a complete case. Under these conditions, recent methodological work has shown that complete-case analysis with principled covariate adjustment, as conducted here through DAG-guided sequential models, can yield unbiased estimates even when data are not missing at random, provided that missingness is neither outcome-affected nor mediator-affected [56,57].

Although estimates for oral functional-status categories were directionally consistent across sequential models, effect magnitudes may be unstable given the limited number of deaths. Furthermore, oral health status was assessed at a single time point, precluding evaluation of changes in dentition or prosthesis use over follow-up, as well as the absence of location of remanent dentition to establish functional occlusal units [58]. Similarly, prostheses use was self-reported and did not capture clinical and patient-related outcomes such as prosthesis quality, fit, or functional effectiveness, which may have led to residual heterogeneity within exposure categories and its impact on mortality [59].

Additionally, frailty components partly relied on self-reported measures, which may be subject to reporting bias. However, frailty was assessed using a validated modified phenotype and included objective components such as handgrip strength and measured anthropometry, limiting the potential impact of misclassification [60]. Furthermore, frailty status was dichotomized (pre-frail and frail versus robust) for the regression analyses, which may have attenuated the association between frailty and mortality by collapsing clinically distinct categories. Additionally, given the exploratory nature of the sensitivity structural model and the limited number of events, these results should be interpreted as supportive rather than confirmatory. To be noted, residual confounding cannot be excluded. Unmeasured factors such as lifetime socioeconomic trajectories, social interactions [61], dietary quality, inflammatory markers, or access to rehabilitative dental care may partially explain the observed associations [9]. Finally, the lack of clear associations between frailty, diabetes, or hypertension and mortality after adjustment may reflect limited power, collinearity with age, or the possibility that oral functional status captures aspects of cumulative health vulnerability not fully represented by these clinical diagnoses.

Despite these limitations, the observed associations support continued investigation of oral functional status within healthy ageing frameworks. The findings are consistent with the view that oral health may reflect broader patterns of cumulative vulnerability in later life, and they reinforce the value of integrating oral health measures into population-based ageing research.

## 5. Conclusions

In this exploratory linked survey analysis, oral functional status (based on dentition and prosthesis use) was associated with all-cause mortality among older adults in Chile. However, the modest analytic sample size, limited number of deaths, and wide confidence intervals constrain the precision of the estimates. These findings are hypothesis-generating and are compatible with oral functional status as a marker of broader health vulnerability in ageing. They do not establish causality and should be confirmed in larger cohorts with standardized outcome definitions, repeated oral assessments, and sufficient event counts for stable multivariable modelling and interaction testing. In particular, future studies should explore potential effect modification between oral functional status and frailty on mortality, as the hypothesis that tooth loss is a marker of cumulative physiological decline suggests that these two domains may interact in shaping survival trajectories.

## Figures and Tables

**Table 1:** Characteristics of the study sample, participants aged 60 years and over.

Variable	Total (n = 223, N = 1,016,557)	Individuals with <10 Teeth Without Prostheses (n = 10)	Individuals with <10 Teeth Who Wore Prostheses (n = 109)	Individuals with ≥10 Teeth (≥5 per Arch) Without Prostheses (n = 60)	Individuals with ≥10 Teeth (≥5 per Arch) Who Wore Prostheses (n = 44)	*p* ^a^
Age [mean (95% CI)]	69.63 (68.02, 71.23)	74.46 (68.74, 76.18)	72.93 (70.41, 75.46)	66.37 (64.12, 69.21)	67.44 (64.82, 70.07)	<0.05 ^b^
Sex [% (95% CI)]						
Male	51.24 (43.47, 58.95)	45.52 (14.54, 80.41)	40.22 (27.22, 54.75)	64.76 (50.17, 77.03)	51.71 (33.23, 69.74)	>0.1
Female	48.76 (41.05, 56.53)	54.48 (19.59, 85.46)	59.78 (45.25, 72.78)	35.2 (22.97, 49.83)	48.29 (30.26, 66.77)	
Educational attainment [% (95% CI)]						
<8 years	36.23 (27.14, 46.41)	47.59 (15.37, 81.95)	60.32 (44.3, 74.39)	16.64 (8.27, 30.63)	18.3 (8.55, 34.95)	<0.01
8–12 years	41.12 (31.22, 51.79)	39.11 (10.06, 78.67)	30.01 (19.69, 42.86)	38.76 (23.4, 56.74)	68.3 (49.31, 82.67)	
≥13 years	22.66 (14.32, 33.93)	13.3 (1.71, 57.51)	9.67 (2.86, 27.99)	44.6 (27.17, 63.47)	13.4 (5.21, 30.35)	
Area of residence [% (95% CI)]						
Urban	99.36 (97.95, 99.81)	100	99.35 [99.12, 99.51]	99.69 [97.72, 99.96]	98.76 [91.34, 99.83]	>0.1
Rural	0.63 (0.19, 2.05)	-	0.65 [0.49, 0.88]	0.31 [0.04, 2.28]	1.24 (0.17, 8.66)	
Frailty [% (95% CI)]						
Robust	34.13 (26.62, 42.53)	0.47 (0.05, 3.89)	33.94 (22.62, 47.46)	41.82 (27.92, 57.15)	27.09 (14.09, 45.70)	>0.1
Pre-frail	56.53 (48.20, 64.50)	72.42 (32.01, 93.61)	54.49 (41.32, 67.07)	50.30 (34.22, 66.33)	68.65 (49.73, 82.90)	
Frail	9.34 (5.70, 14.95)	27.11 (6.16, 67.83)	11.56 (5.70, 22.05)	7.88 (2.71, 20.80)	4.26 (0.58, 25.47)	
Diabetes [% (95% CI)]						
Yes	28.26 (20.24, 37.93)	43.60 (12.57, 80.61)	31.50 (19.78, 46.17)	17.61 (8.60, 32.69)	37.20 (20.07, 58.28)	>0.1
No	71.74 (62.07, 79.76)	56.40 (19.39, 87.43)	68.50 (53.83, 80.22)	82.39 (67.31, 91.40)	62.80 (41.72, 79.93)	
Hypertension [% (95% CI)]						
Yes	66.03 (56.37, 74.52)	86.41 (41.88, 98.25)	77.52 (63.82, 87.08)	56.35 (37.42, 73.59)	55.52 (37.32, 72.36)	>0.05
No	33.97 (25.48, 43.63)	13.59 (1.75, 58.12)	22.48 (12.92, 36.18)	43.65 (26.41, 62.58)	44.48 (27.64, 62.68)	

^a^ Chi-square test. ^b^ ANOVA test. CI: Confidence Interval, SD: Standard Deviation.

**Table 2:** Causes of death according to ICD-10 categories and subcategories.

Category (% of Deaths)	ICD-10 Code	Cause of Death (Description)	n
Neoplasms (38.5%)	C16.9	Malignant neoplasm of stomach, unspecified	2
	C18.9	Malignant neoplasm of colon, unspecified	1
	C22.1	Intrahepatic bile duct carcinoma	1
	C22.9	Malignant neoplasm of liver, unspecified	3
	C25.9	Malignant neoplasm of pancreas, unspecified	2
	C26.9	Malignant neoplasm of digestive organ, unspecified	1
	C34.9	Malignant neoplasm of bronchus and lung, unspecified	3
	C49.9	Malignant neoplasm of connective and soft tissue, unspecified	1
	C61	Malignant neoplasm of prostate	1
Cardiovascular diseases (17.9%)	I11.0	Hypertensive heart disease with heart failure	1
	I21.9	Acute myocardial infarction, unspecified	1
	I25.1	Atherosclerotic heart disease	1
	I50.9	Heart failure, unspecified	1
	I61.9	Intracerebral hemorrhage, unspecified	1
	I67.8	Other specified cerebrovascular diseases	1
	I69.4	Sequelae of stroke, not specified as hemorrhage or infarction	1
Respiratory diseases (25.6%)	J18.9	Pneumonia, unspecified organism	1
	J69.0	Pneumonitis due to food and vomit	1
	J84.1	Other interstitial pulmonary diseases with fibrosis	2
	U07.1	COVID-19, virus identified	6
Neurological disorders (5.1%)	F03	Unspecified dementia	1
	G30.9	Alzheimer disease, unspecified	1
Digestive diseases (2.6%)	K80.3	Calculus of gallbladder with cholecystitis	1
Symptoms and ill-defined conditions (2.6%)	R57.8	Other shock	1
Injury-related causes (7.7%)	S25.0	Injury of thoracic aorta	1
	S72.9	Fracture of femur, unspecified	1
	T59.8	Toxic effect of other gases, fumes and vapours	1
Total			39

**Table 3:** Sequential survey-weighted Cox models for mortality.

Variables	Model 1	Model 2	Model 3	Model 4	Model 5
<10 teeth + denture vs. <10 teeth (no denture)	0.20 (0.05–0.80); *p* = 0.023	0.19 (0.05–0.77); *p* = 0.021	0.17 (0.05–0.61); *p* = 0.007	0.28 (0.08–0.96); *p* = 0.043	0.27 (0.09–0.84); *p* = 0.024
≥10 teeth (no denture) vs. <10 teeth (no denture)	0.18 (0.04–0.94); *p* = 0.042	0.17 (0.03–0.89); *p* = 0.036	0.16 (0.04–0.74); *p* = 0.019	0.14 (0.03–0.67); *p* = 0.014	0.13 (0.03–0.65); *p* = 0.014
≥10 teeth + denture vs. <10 teeth (no denture)	0.12 (0.02–0.65); *p* = 0.014	0.11 (0.02–0.75); *p* = 0.025	0.08 (0.01–0.46); *p* = 0.005	0.10 (0.02–0.65); *p* = 0.016	0.09 (0.02–0.42); *p* = 0.002
Female (vs. male)	0.92 (0.44–1.91); *p* = 0.823	0.97 (0.39–2.40); *p* = 0.952	0.79 (0.29–2.17); *p* = 0.644	—	—
Age (per year)	1.12 (1.07–1.18); *p* < 0.001	1.12 (1.07–1.18); *p* < 0.001	1.15 (1.09–1.20); *p* < 0.001	—	—
Education: 8–12 years (vs. 0–7)	1.29 (0.51–3.25); *p* = 0.589	1.24 (0.48–3.22); *p* = 0.657	1.51 (0.54–4.23); *p* = 0.425	—	—
Education: ≥13 years (vs. 0–7)	1.56 (0.44–5.55); *p* = 0.490	1.50 (0.45–5.04); *p* = 0.510	1.74 (0.56–5.42); *p* = 0.336	—	—
Rural area (vs. urban)	3.44 (1.14–10.39); *p* = 0.029	3.32 (1.17–9.43); *p* = 0.024	3.92 (1.29–11.98); *p* = 0.017	—	—
Frailty (yes vs. no)	—	0.81 (0.17–3.80); *p* = 0.782	1.04 (0.23–4.61); *p* = 0.962	1.25 (0.36–4.26); *p* = 0.725	—
Type 2 diabetes (yes vs. no)	—	—	1.82 (0.80–4.13); *p* = 0.150	—	1.04 (0.38–2.86); *p* = 0.934
Hypertension (yes vs. no)	—	—	0.48 (0.18–1.27); *p* = 0.137	—	0.94 (0.33–2.69); *p* = 0.911

## Data Availability

The data that support the findings of this study are available from the corresponding author upon reasonable request. However, the dataset from the Chilean NHS 2016–2017 is publicly available in https://epi.minsal.cl/condiciones-de-uso/ (accessed on 17 January 2026).

## Abbreviations

The following abbreviations are used in this manuscript:

BMI	Body mass index
CI	Confidence interval
COVID-19	Coronavirus disease 2019
DAG	Directed acyclic graph
GPAQ	Global Physical Activity Questionnaire
HR	Hazard ratio
ICD-10	International Classification of Diseases, 10th Revision
MD	Minimum dentition
MET	Metabolic equivalent of task
NHS	National Health Survey
STROBE	Strengthening the Reporting of Observational Studies in Epidemiology
T2DM	Type 2 diabetes mellitus

## Appendix A

**Table A1:** Parameter estimates from the multivariable joint survival model using generalized structural equation modelling.

Equation	Predictor	β	SE	t	*p*-Value	95% CI
Time-to-event (mortality)	Geographic area	1.385	0.721	1.92	0.057	−0.043 to 2.812
	Frailty status (binary)	0.223	0.628	0.36	0.723	−1.021 to 1.467
	Age (years)	0.123	0.025	4.95	<0.001	0.074 to 0.172
	Sex	−0.358	0.428	−0.83	0.406	−1.206 to 0.491
	Dental prosthesis status	−0.646	0.355	−1.82	0.071	−1.350 to 0.057
	Diabetes	0.552	0.393	1.41	0.163	−0.226 to 1.329
	Hypertension	−0.629	0.419	−1.50	0.136	−1.460 to 0.202
	Educational attainment	0.374	0.324	1.15	0.251	−0.269 to 1.017
Dental prosthesis status	Geographic area	0.537	0.570	0.94	0.348	−0.593 to 1.667
	Age (years)	−0.032	0.011	−3.07	0.003	−0.053 to −0.011
	Sex	−0.094	0.194	−0.49	0.628	−0.479 to 0.290
	Diabetes	0.028	0.249	0.11	0.912	−0.466 to 0.521
	Educational attainment	0.331	0.146	2.27	0.025	0.042 to 0.621
Diabetes	Geographic area	−0.645	1.764	−0.37	0.715	−4.140 to 2.850
	Age (years)	−0.043	0.029	−1.46	0.147	−0.101 to 0.015
	Sex	0.165	0.431	0.38	0.703	−0.690 to 1.020
	Educational attainment	−0.747	0.298	−2.51	0.013	−1.337 to −0.158
Hypertension	Geographic area	−0.417	0.869	−0.48	0.632	−2.139 to 1.304
	Age (years)	0.084	0.025	3.34	0.001	0.034 to 0.134
	Sex	0.131	0.398	0.33	0.743	−0.658 to 0.920
	Educational attainment	−0.103	0.258	−0.40	0.690	−0.615 to 0.409
Frailty status (binary)	Age (years)	0.042	0.030	1.42	0.158	−0.017 to 0.101
	Sex	1.257	0.712	1.76	0.080	−0.154 to 2.667
	Dental prosthesis status	−0.109	0.423	−0.26	0.798	−0.947 to 0.730
	Diabetes	−0.835	0.790	−1.06	0.293	−2.400 to 0.730
	Hypertension	−0.660	0.594	−1.11	0.269	−1.836 to 0.516
	Educational attainment	−1.167	0.444	−2.63	0.010	−2.047 to −0.287
Ancillary parameter (/t)	Weibull shape parameter	0.794	0.223	—	—	0.352 to 1.235

SE = standard error; CI = confidence interval. Coefficients are reported from the fitted joint parametric survival model with linearized standard errors. The time-to-event equation is parameterized with a Weibull distribution (ancillary parameter ln p).
